# Migration patterns of *Gentiana crassicaulis*, an alpine gentian endemic to the Himalaya–Hengduan Mountains

**DOI:** 10.1002/ece3.8703

**Published:** 2022-03-18

**Authors:** Lianghong Ni, Weitao Li, Zhili Zhao, Dorje Gaawe, Tonghua Liu

**Affiliations:** ^1^ 66322 Shanghai University of Traditional Chinese Medicine Shanghai China; ^2^ Tibetan Traditional Medical College Lhasa China; ^3^ 47839 Beijing University of Chinese Medicine Beijing China

**Keywords:** genetic divergence, *Gentiana crassicaulis*, phylogeography, Qinghai–Tibetan Plateau

## Abstract

The Himalaya–Hengduan Mountain region is one of the hotspots of biodiversity research. The uplift of the Qinghai–Tibetan Plateau (QTP) and the Quaternary glaciation caused great environmental changes in this region, and the responses of many species in the QTP to the Quaternary climate are still largely unknown. The genetic structure and phylogeographical history of *Gentiana crassicaulis* Duthie ex Burk, an endemic Chinese alpine species in this area, were investigated based on four chloroplast fragments and internal transcribed spacer region of the nuclear ribosomal DNA (nrITS) sequences of 11 populations. The populations with highly diverse chloroplast haplotypes were mainly found at the edge of the QTP. There were two main haplotypes of nrITS clones, one shared by the Yunnan and Guizhou populations, and the other by the remaining populations. The population with the highest diversity was the Gansu population, located at the edge of the plateau. Based on molecular dating, the diversification of *G. crassicaulis* at the edge of the plateau occurred before the Last Glacial Maximum (LGM), and the species may have completed its expansion from the edge to the platform. Ecological niche models were conducted to predict the distributional ranges of *G. crassicaulis* at present, during the LGM, and during the last interglacial (LIG) period. The results demonstrated that *G. crassicaulis* survived on the QTP platform and at the edge during the LGM but afterward retreated from the platform to the southern edge, followed by expansion to the platform.

## Graphical abstract

*Gentiana crassicaulis*, an alpine gentian endemic to the Himalaya–Hengduan Mountains, could survive on the QTP platform and at the edge during the LGM but afterward retreated from the platform to the southern edge, followed by expansion to the platform.

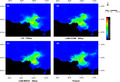

## 1 INTRODUCTION

The Himalayan–Hengduan Mountain region is one of the global biodiversity hotspots and refugia of many plants in Eurasia (Myers et al., 2000; Sechrest et al., 2000). Due to its complex geological history and climatic conditions, this region has developed rich species diversity and is an ideal area for studying the mechanisms of speciation. The uplift of the Qinghai–Tibetan Plateau (QTP) and the Quaternary glaciers caused great environmental changes in this region. These changes not only altered the distribution patterns of plants but also changed the genetic structures of many species. Studies on the differentiation, adaptive evolution, and migration patterns of the plant species since the Quaternary period in this region have been carried out gradually (Li, Zhai, et al., 2011; Luo et al., 2016). By analyzing the DNA variation among different populations of a species and the phylogeographic information of these populations, the population dispersal and migration routes of that species can be evaluated, whereby the historical causes of the current distribution patterns can be predicted. In particular, this strategy can be used to reveal the distribution patterns of plants and the possible glacial refugia in the Himalaya–Hengduan Mountains.

*Gentiana* L. is the largest genus of Gentianaceae and widely distributed in the temperate alpine regions of Europe, Asia, and North America. The QTP region in southwest China is the distribution center of the genus and also the region with the most abundant species diversity and endemic distribution of the genus. Sect. *Cruciata* Gaudin is a section of *Gentiana*, containing approximately 20 species worldwide (Ho & Liu, 2001). *Gentiana crassicaulis* Duthie ex Burk. is a species in sect. *Cruciata* Gaudin. This species is endemic to the Himalaya–Hengduan Mountains and distributed over southeastern Tibet, Yunnan, Sichuan, northwestern Guizhou, southeastern Qinghai, and southern Gansu of China (He, 1988). Radiative speciation exists in Sect. *Cruciata*, and many species in Sect. *Cruciata* experienced rapid species differentiation along with the uplift of the QTP and the drastic changes in the climate and environment (Zhang et al., 2009), making *G. crassicaulis* an ideal research object for the analysis of species distribution patterns in the Himalaya–Hengduan Mountain region.

In recent years, there have been many studies on the phylogeography of the alpine plants in the QTP. Based on related species, Qiu et al. have proposed that the genealogical history of the plants in the QTP mainly has two forms (Qiu et al., 2011). (1) In the Quaternary glacial period, the populations on the platform of the plateau retreated to the edge of the plateau—the east and southeast areas with lower elevations than the platform. During the interglacial period and latter glacial period, the plants moved back from the refugia at the edge of the plateau to the platform. The genetic diversity of the plant species, such as *Juniperus przewalskii* (Li et al., 2011), *Sibiraea angustata* (Duan et al., 2011), *Metagentiana striata* (Chen et al., 2008), *Spiraea Mongolica* (Khan et al., 2018), and *Bupleurum smithii* (Zhao et al., 2013), is high in the populations on the plateau edge. (2) In the Quaternary glacial period, the populations on the platform of the plateau retreated to the local refugia and retained the ancient haploids and diversity. In the late interglacial period, the plant species, such as *Juniperus tibetica* complex (Opgenoorth et al., 2010), *Rhodiola* sect. *Trifida* (Gao et al., 2016; Li et al., 2018), *Meconopsis integrifolia* (Yang et al., 2012), and *Potentilla fruticosa* (Sun et al., 2010), spread around from the local refugia. Each species has either of these two forms of genealogical history, with species‐specific characteristics. Some species, such as *Paeonia delavayi* and *P. ludlowii*, have a "dual pattern" of both in situ retention and retreat to the refugia (Zhang et al., 2018). In general, populations in a refugium have higher genetic diversity and more unique haplotypes than populations formed through migration (Hewitt, 2000). A phylogeographic study has revealed that *Gentiana straminea* Maxim., another species of sect. *Cruciata*, survived on the QTP platform and gradually extended to the edge of the platform during the Last Glacial Maximum (LGM), showing a different phylogeographic pattern from those of most alpine plants previously reported (Lu et al., 2015).

Ecological niche models (ENMs) are a class of methods to predict the actual and potential distribution of species based on geographical distribution and related environmental factors. ENMs have been widely used in spread of invasive species (Thuiller et al., 2005), conservation biology (Graham et al., 2004), impact of climate change (Li et al., 2021), and phylogeography (Khan et al., 2018). With the rapid development of Geographic Information Systems technology, ENMs have developed rapidly. As one of the most commonly employed models, MaxEnt uses the principle of maximum entropy on presence‐only data to estimate a set of functions that relate environmental variables and habitat suitability in order to approximate the species’ niche and potential geographic distribution (Merow et al., 2013). Hernandez et al. (2006) constructed ENM for 18 species in California with four different methods (Bioclim, Domain, GARP, and MaxEnt), and the results showed that the model constructed by MaxEnt performed best. Wisz et al. (2008) evaluated ENM constructed by 46 organisms (including alpine plants) using 12 methods and also found that the result of MaxEnt was superior to those of the other 11 methods. MaxEnt Model has been widely used in studies similar to this one. For example, phylogeography of *Rhodiola* sect. *Trifida* (Crassulaceae) revealed multiple microrefugia on the QTP and supported the hypothesis that species with similar geographic distribution and inhabiting the same community had similar responses to the Quaternary climatic oscillations (Li et al., 2018). MaxEnt model also played an important role in study of phylogeography of *Parasyncalathium souliei* (Asteraceae) and its potential application in delimiting phylogeoregions in the QTP–Hengduan Mountains hotspot (Lin et al., 2018).

Previous studies suggest that the herbaceous plants flourished in China from the Miocene of tertiary (Tao, 1992). In the late Miocene, some typical genera with wide distribution in QTP, such as *Gentiana*, *Metagentiana*, and *Primula*, underwent strong radiative differentiation, resulting in more new species (Axelrod et al., 1996). Does the speciation and differentiation of *G. crassicaulis* conform to the above situation? *G. crassicaulis* and *G. straminea* are related species of Sect. *Cruciata*, and they are both cold‐tolerant perennials with overlapping distribution areas. Does *G. crassicaulis* show a different genealogical geography from most alpine plants, as does *G. straminea*, or is there a specific type of *G. crassicaulis*? These questions are worthy of discussion and can provide references for further analysis of the glacial history of alpine plants in *Gentiana*. This study evaluated the genetic diversity and phylogeographical structure of *G. crassicaulis* by using internal transcribed spacer (ITS) cloning sequences, four chloroplast fragments, and MaxEnt models in three periods (during the last interglacial (LIG) period, during the LGM, and at present) to reveal the species differentiation and migration patterns, with the following purposes: (1) to evaluate the value of chloroplast and ITS data in the systematic analysis of *G. crassicaulis*, (2) to evaluate the genetic differentiation level of *G. crassicaulis*, and (3) to investigate how this species expanded and contracted its range during the Quaternary glacial and interglacial periods.

## 2 MATERIALS AND METHODS

### 2.1 Plants

For this study, 113 individuals from 11 populations of *Gentiana crassicaulis* Duthie ex Burk were collected throughout the distribution range, with sample size per population ranging from 3 to 15 depending on the population size. The individuals collected were ≥5 m apart to avoid sampling bias toward closely related individuals (see Table S1 for the detailed information about the samples). Fresh leaves were taken from each sample and quickly dried using a silica gel. The voucher specimens were deposited in the herbarium of the School of Pharmacy, Shanghai University of Traditional Chinese Medicine, China.

### 2.2 Chloroplast genome sequencing

Chloroplast genomes of *G. crassicaulis* from eight populations were sequenced and annotated as previously reported (Ni et al., 2017; Wei et al., 2018). The sequences were analyzed, and four fragments with variable sites among the populations were selected for analysis.

### 2.3 DNA extraction, amplification, and sequencing

Total genomic DNA was extracted from the silica gel–dried leaves by using the modified CTAB method (Doyle & Doyle, 1987). Four chloroplast fragments were amplified using the following primers: *rpl*33, 5′‐GCAGGTCTATTGATAGAGATTAATCG‐3′ and 5′‐CCAGCAGTTCTAGTGGTCGACTCGGTT‐3′; *rpl*33‐*rps*18, 5′‐GCCAATCGGGGGATCGAATTGATTATAG‐3′ and 5′‐GAATTAAACGAGGATATATAGCTCGG‐3′; *rpl*16 intron, 5′‐TCCCGAAATAATGAATTGAGTTCG‐3′ and 5′‐TCAGAGAAGGTAGGGTTCCCCT‐3′; and *trn*C‐GCA‐*pet*N, 5′‐GGCGGCATGGCCGAGTGGTAAGGC‐3′ and 5′‐TCCACTTCTTCCCCATACTACGA‐3′. The cycling profile was as follows: initial denaturation at 94°C for 5 min, followed by 42 cycles of 30 s at 94°C, 40 s of annealing at 48–60°C, 1–3 min of elongation at 72°C, and ending with a 7 min extension at 72°C. The four chloroplast fragments were concatenated for the analysis.

The ITS region was amplified using the PCR conditions previously reported (Xiong et al., 2015). There were numerous ambivalent peaks in the direct sequencing chromatogram. Thus, the PCR products were cloned using the pUCm‐T Vector PCR Products Cloning kit (Sangon, Shanghai), and 10° clones per individual were sequenced (Yuan & Philippe, 1995).

### 2.4 Sequence analysis

The sequences were aligned using MEGA7 (Tamura et al., 2013). For each sequence dataset, DNASP5 (Rozas et al., 2003) was used to estimate the number of haplotypes (*n*), haplotype diversity (*Hd*), nucleotide diversity (π), average number of nucleotide differences (*k*), gene flow (*Nm*), and genetic differentiation (*Fst*). The mismatch distribution and neutrality tests (Tajima's D) were performed using DNASP5. Total gene diversity (*Ht*) and average gene diversity within populations (*Hs*) were calculated. To test whether there was a phylogeographic structure, *G_ST_* and *N_ST_* were calculated using PERMUT (Pons & Petit, 1996). Differentiation within and among populations, and *F_st_* were calculated using analyses of molecular variance (AMOVA) (Excoffier et al., 1992) to estimate genetic variation by using ARLEQUIN 3.1 (Excoffier et al., 2005), with the significance tested using 10,000 permutations. Haplotype networks were constructed using NETWORK v4.2.0.1 (Bandelt et al., 1999).

### 2.5 Phylogenetic analysis

Phylogenetic analysis was performed based on chloroplast and ITS haplotypes. Maximum‐parsimony (MP) analysis was conducted using PAUP 4.0 (Swofford, 2002), with all characters considered equally weighted and with gaps treated as missing data. Heuristic searches were replicated 1000 times with random taxon addition sequences, TBR branch swapping, and with the options Multrees and Steepest Descent in effect. The bootstrap analyses used 1000 replicates with 10 replicates of random addition sequences and branch swapping, and then a maximum of 1000 trees of the random addition replicates were saved. The phylogenetic trees were viewed and adjusted using FigTree1.4.3.

### 2.6 Estimation of divergence time

BEAST v2.0.1 (Drummond et al., 2012) was used to infer the divergence time between lineages. Chloroplast datasets were used to conduct dating analysis with an uncorrelated lognormal relaxed molecular clock model, as reported by Li et al. (2018). Because there were no fossil records reported of sect. *Cruciata* or their close relatives, we used substitution rates to estimate the divergence times. The substitution rate of chloroplast sequence varies from 1.0 × 10^−9^ to 3.0 × 10^−9^ s s^−1^ year^−1^ (Wolfe et al., 1987).

### 2.7 Ecological niche modeling

We used ecological niche models (ENMs) based on the maximum entropy method implemented in MAXENT Version 3.4.0 (Phillips & Dudík, 2008) to predict the distributional ranges of *G. crassicaulis* at present, during the LGM (ca. 20 kilo years ago, kya) (MIROC Model; CCSM Model), and during the last interglacial (LIG, ca. 130 kya) period (Di Pasquale et al., 2020; Li et al., 2018, 2021; Lu et al., 2015). Two different evaluation procedures were used for accuracy assessment of the model outputs: (1) a threshold‐independent statistic—the area under the receiver operating curve (AUC), and (2) a threshold‐dependent statistic—the true skill statistic (TSS). The AUC is calculated using a number of randomly selected sites equal to the number of test presence sites and provides a quantitative measure of model performance that ranges between 0 and 1, with values close to 1 indicating a reliable fit (Phillips et al., 2006). The TSS is measured as: TSS = Sensitivity + Specificity − 1. TSS scores vary between +1 and −1, with a score close to 1 indicating an almost perfect model, while close to 0 or less than 0 indicates a model no better than random (Allouche et al., 2006; Monserud & Leemans, 1992). The AUC value of the MaxEnt model in each period was >0.97, and TSS value was >0.76, indicating high stability and accuracy (Table S2). The localities of *G. crassicaulis* were identified from the field collections by the authors or from the online herbarium records (e.g., Global Biodiversity Information Facility and Chinese Virtual Herbarium). The data obtained from online databases were double checked. A total of 79 spatially unique localities were used for the analysis (Table S3). Nineteen environmental variables for each period were retrieved from the WorldClim database (http://worldclim.org) with a resolution of 2.5 arcmin. Multivariate collinearity among climate variables was tested by Pearson correlation coefficient using SPSS 20.0. The climate variables with Pearson correlation coefficient less than 0.8 were retained, and among the variables with Pearson correlation coefficient greater than 0.8, the one which was closely related to geographical distribution was selected. Eight climate variables were screened out and used (Zhang et al., 2020) (Table S4). The distributions were estimated, with the settings for convergence threshold (10^−5^), number of iterations (500), and occurrence localities divided into testing and training datasets (25% and 75%, respectively).

## 3 RESULTS

### 3.1 Chloroplast genomes

**FIGURE 1:**
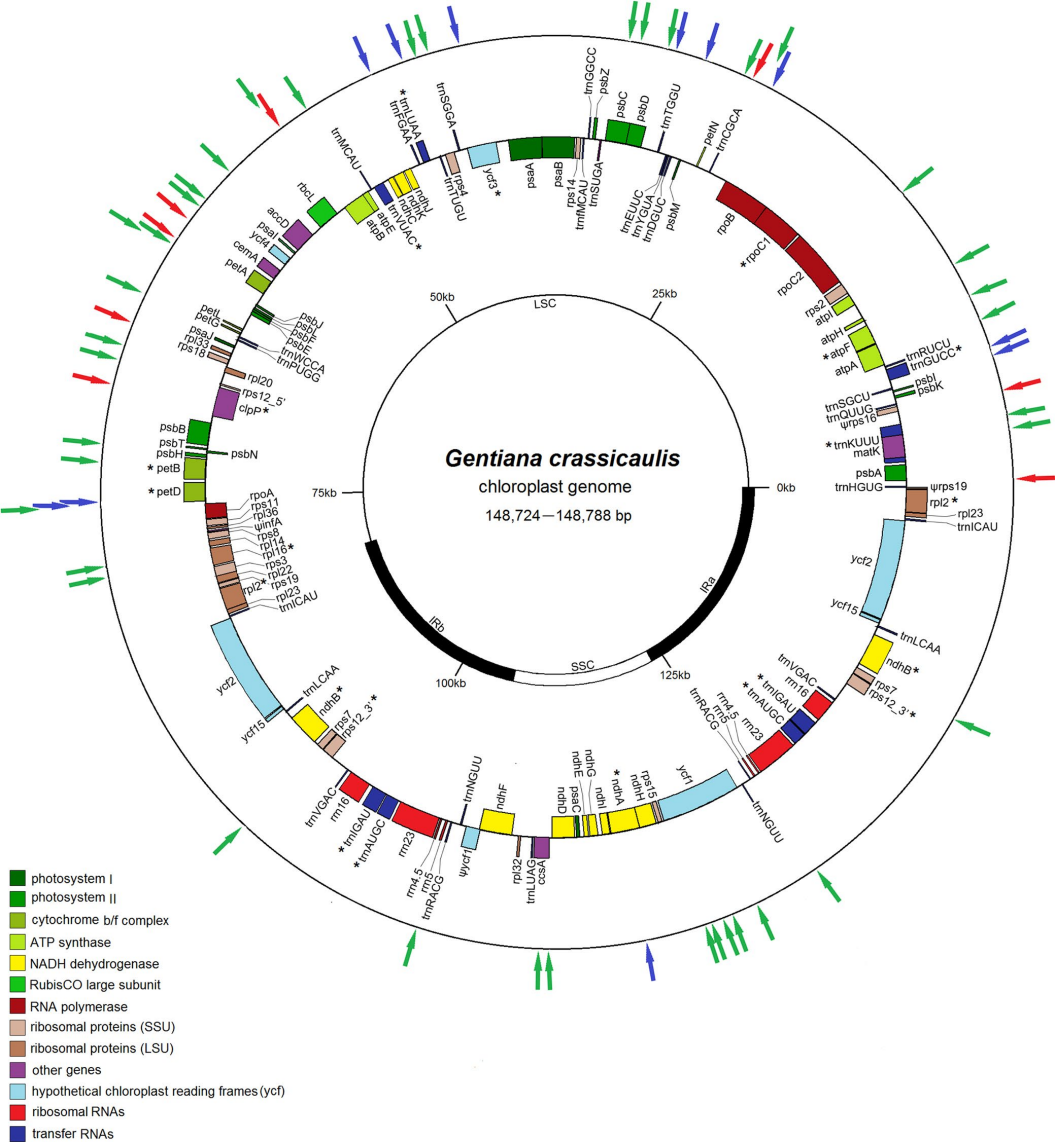
Chloroplast genome map of *Gentiana crassicaulis*. The genes drawn inside the circle are transcribed clockwise, and those outside counterclockwise. The genes belonging to different functional groups are shown in different colors. The asterisks indicate the genes that contain intron(s). The red, green, and blue arrows indicate the indels, SNPs, and SSRs with difference, respectively

Chloroplast genome sequences, ranging from 148,724 to 148,788 bp, of eight *G. crassicaulis* populations were obtained. A total of 56 variable sites, including 37 single nucleotide polymorphisms (SNPs), 11 simple sequence repeat (SSR) differences, and 8 insertion–deletions (InDels) were discovered (Figure 1). The detailed information about the chloroplast genomes is shown in Table S5. Four fragments with variable sites were selected for phylogeographical analysis.

### 3.2 Chloroplast DNA variation, geographical distribution, and phylogenetic relationships

Four chloroplast fragments, including *rpl33*, *rpl33*‐*rps18*, *rpl16* intron, and *trnC*‐*GCA*‐*petN*, from 113 individual plants from 11 populations of *G. crassicaulis*, were amplified and sequenced (Table S6). The four fragments were concatenated, and a total of six haplotypes (H1–H6) were identified (Table 1). These haplotypes ranged from 2040 to 2058 bp, and the length after alignment was 2066 bp.

**TABLE 1:** Variable sites in the aligned sequences of four chloroplast fragments from six haplotypes of *Gentiana crassicaulis*

	*rpl*16 intron^a^	*rpl*33^a^	*rpl*33‐*rps*18^a^	*trn*C‐GCA‐*pet*N^a^	
Haplotype	167^b^	176^b^	58^b^	129^b^	291^b^
H1	G	C	GAAGTAAGG	TTTTTCAA	C
H2	A	C	GAAGTAAGG	–	C
H3	A	C	GAAGTAAGGGAAGTAAGG	–	C
H4	G	C	–	–	T
H5	G	A	GAAGTAAGG	–	C
H6	G	C	GAAGTAAGG	–	C

–, Deletion.

^a^ Chloroplast fragment.

^b^ Site.

**FIGURE 2:**
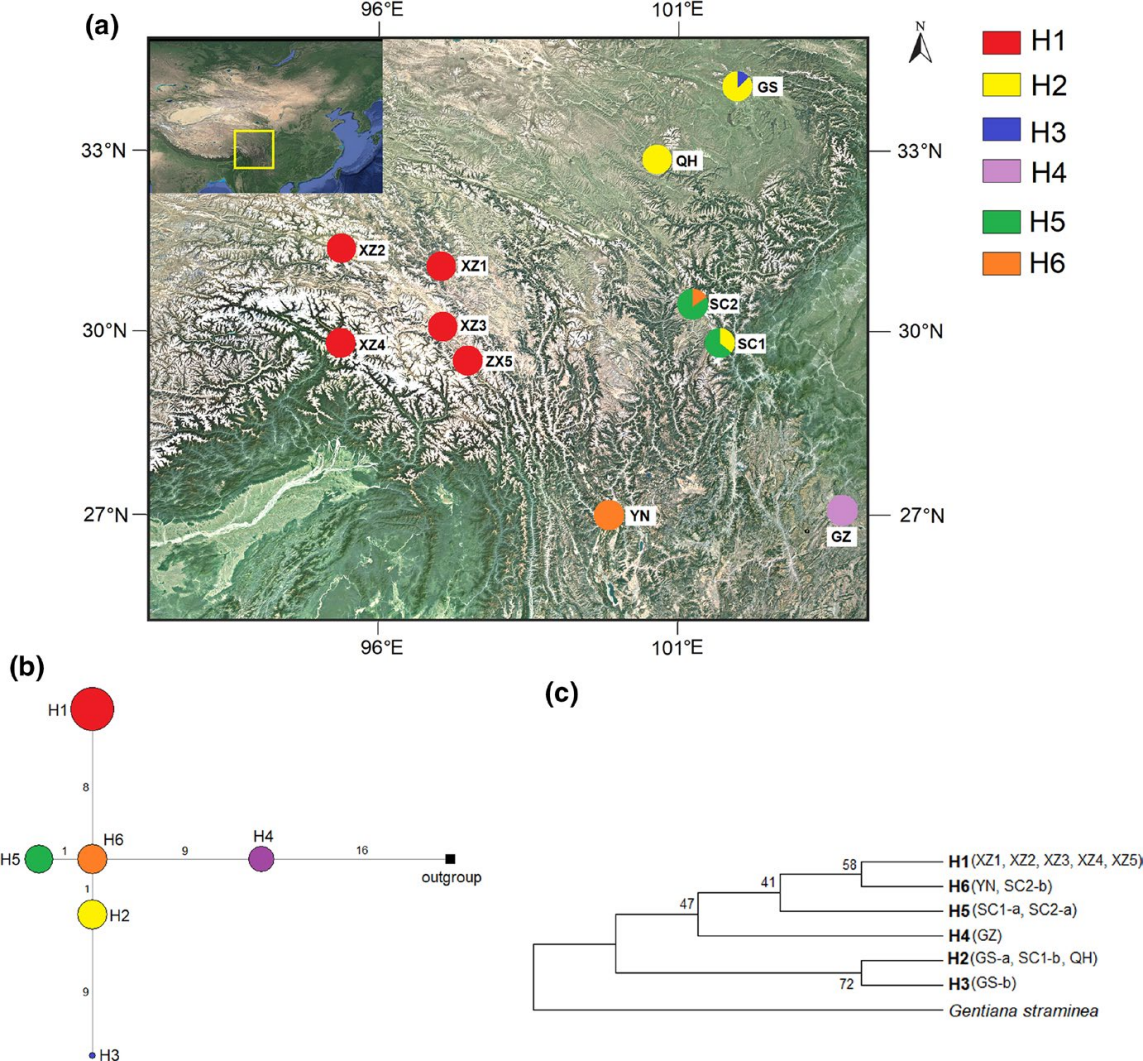
(a) Geographical distributions of the chloroplast haplotypes of *Gentiana crassicaulis*. The pie charts show the proportions of haplotypes within each population. (b) Network of the chloroplast haplotypes detected in *G. crassicaulis*, with *G. straminea* as an outgroup. The sizes of the circles are proportional to the sequence numbers of the haplotypes, with the smallest circle representing *n* = 1. The numbers between two haplotypes represent the corresponding mutational steps. (c) Phylogenic tree based on the chloroplast haplotypes, with *G. straminea* as an outgroup. The numbers are the maximum‐parsimony bootstrap values

H1 was present in five populations (XZ1, XZ2, XZ3, XZ4, and XZ5) in Tibet and was the most widely distributed haplotype. H2 was found in QH (Qinghai), GS (Gansu), and SC1 (Sichuan, Kangding) populations. H5 was detected in SC1 and SC2 (Sichuan, Daofu) populations. H6 was present in YN (Yunnan) and SC2 populations. H3 and H4 were private haplotypes, which were found to exist only in GS and GZ (Guizhou) populations, respectively. GS, SC1, and SC2 populations each had two haplotypes, and there was only one haplotype per population in the other populations. The geographical distribution of each haplotype is shown in Figure 2a.

A network of *G. crassicaulis* was constructed based on the chloroplast haplotypes, with *Gentiana straminea* as an outgroup (Figure 2b). The results showed that H4 was adjacent the outgroup and then connected with H6 and H1. H5 and H2 were connected with H6 to form a large branch, and H3 was derived from H2.

An MP phylogenetic tree was constructed based on the chloroplast haplotypes, with *Gentiana straminea* as an outgroup (Figure 2c). The six haplotypes formed two large branches, with H1, H4, H5, and H6 as one branch, and H2 and H3 as the other. However, the support rate of each branch was low, and the phylogenetic tree overall did not solve the relationship among all the haplotypes. Nevertheless, H4 is likely relatively archaic.

### 3.3 ITS variation, geographical distribution, and phylogenetic relationships

**FIGURE 3:**
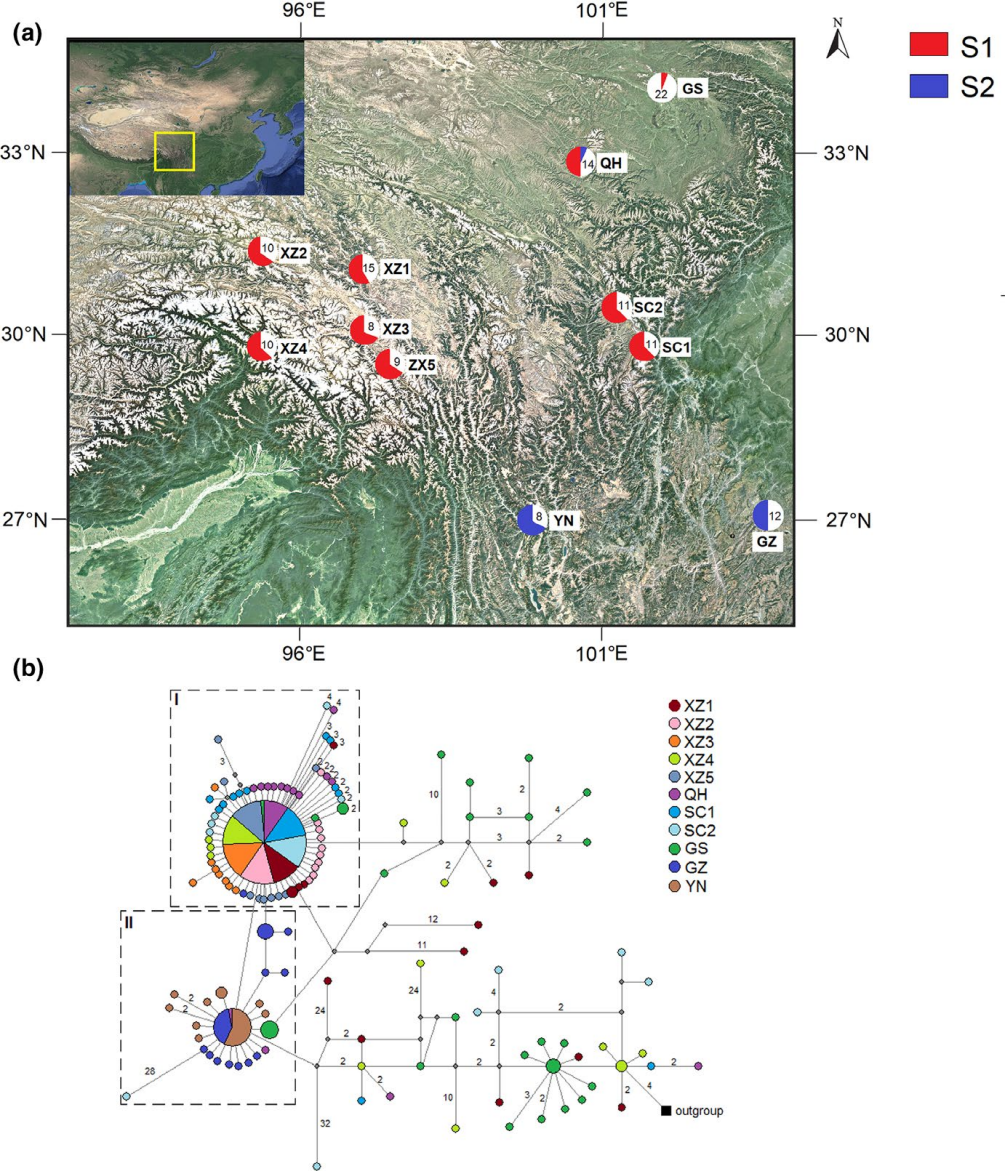
(a) Geographical distribution of the internal transcribed spacer (ITS) haplotypes of *Gentiana crassicaulis*. The pie charts show the proportions of S1 and S2 haplotypes, and the numbers indicate the numbers of the private haplotypes of each population. (b) Network of the ITS haplotypes detected in *G. crassicaulis*, with *G. straminea* as an outgroup. The sizes of the circles are proportional to the sequence numbers of the haplotypes, with the smallest circle representing *n* = 1. The two largest circles indicate the dominant S1 and S2. The putative haplotypes are represented as gray dots on the lines linking two haplotypes. The numbers noted between two haplotypes represent the corresponding mutational steps, with one mutational step not indicated

From 11 populations of *G. crassicaulis*, 330 ITS cloning sequences were obtained (Table S6), and 133 haplotypes (S1–S133) were identified. Of them, 131 haplotypes (98.5%) were private haplotypes unique to a single population. The haplotypes shared among the populations were S1 and S2, which accounted for 44.8% (148/330) and 11.2% (37/330) of all the cloning sequences, respectively. There was only one mutation site (A/G transformation) in the ITS2 region of S1 and S2. S1 was present in XZ1, XZ2, XZ3, XZ4, XZ5, SC1, SC2, QH, and GS populations, and the S1 sequences accounted for >50% in all these populations except in GS population. S2 was detected in QH, YN, and GZ populations. There was only one S2 sequence in QH population, and the S2 sequences in YN and GZ populations accounted for >50%. The geographical distribution of each haplotype and the haplotype of each population are shown in Figure 3a and Figure S1, respectively.

A network of *G. crassicaulis* was constructed based on the ITS haplotypes, with *Gentiana straminea* as an outgroup (Figure 3b). The results showed that all the haplotypes were connected in a network structure, except for a few nodes connected through "putative haplotypes" (haplotypes possibly undetected or extinct). Two main groups were composed of S1 and S2 haplotypes each. Group 1 consisted of S1 and derivative haplotypes, which involved all the populations except YN and GZ populations. Group 2 consisted of S2 and derivative haplotypes, including all the haplotypes of YN and GZ populations and the two haplotypes of GS and SC2 populations. All the other haplotypes showed a dispersed structure. The population containing haplotypes with the highest dispersion was found to be GS population, with haplotypes distributed to different locations of the network. The haplotypes of XZ1 population also showed a dispersed pattern. The haplotypes of XZ2, XZ3, and XZ5 populations were concentrated in Group 1, and the haplotypes of GZ and YN populations were concentrated in Group 2, which is closer than Group 1 to the outgroup.

An MP phylogenetic tree was constructed based on the ITS haplotypes, with *Halenia elliptica* as an outgroup. The results showed that the branches containing S2 involved the haplotypes of GZ and YN populations, and most haplotypes of the other populations showed a dispersed structure. The structure of the phylogenetic tree was similar to that of the Network (Figure S2).

### 3.4 Population genetic diversity and phylogeographic structure

All the populations except GS, SC1, and SC2 were found to have only one chloroplast haplotype. The five populations in Tibet shared the same haplotype, and GZ and GS populations had private haplotypes. The value of *Hd* of each population ranged from 0 to 0.467, lower than the average value of 0.662. The nucleotide diversity (π) value of each population was slightly lower than the average value of 0.040, except for SC1 population (0.046) (Table 2). The average genetic diversity within the populations (*Hs* = 0.086) was much lower than the overall genetic diversity (*Ht* = 0.774). The genetic differentiation coefficient among the populations was high (*Fst* = 0.85605), and the gene flow among the populations was limited (*Nm* = 0.11). There was not a phylogeographic structure for *G. crassicaulis* because *Nst* was not significantly greater than *Gst* (*Nst* = 0.912, *Gst* = 0.889; *p* > .05). AMOVA based on chloroplast haplotypes revealed that 85.61% of the total variation was partitioned among the populations, whereas 14.39% was within the populations (Table 3).

**TABLE 2:** Haplotype composition, number of haplotypes (*n*), haplotype diversity (*Hd*), nucleotide diversity (π), and average number of nucleotide differences (*k*) for the 11 populations of *Gentiana crassicaulis* according to the chloroplast and internal transcribed spacer (ITS) datasets

Pop. ID	Chloroplast DNA				ITS			
	Haplotype composition (number)	*Hd* (SD)	π (×100)	*K*	*n*	*Hd* (SD)	π (×100)	*k*
XZ1	H1(15)	0	0	0	16	0.759 (0.086)	0.948	5.897
XZ2	H1(6)	0	0	0	11	0.563 (0.110)	0.117	0.733
XZ3	H1(3)	0	0	0	9	0.469 (0.114)	0.458	2.860
XZ4	H1(10)	0	0	0	11	0.644 (0.100)	0.821	4.641
XZ5	H1(6)	0	0	0	10	0.517 (0.112)	0.445	2.733
GS	H2 (1), H3 (14)	0.133 (0.112)	0.007	0.133	23	0.968 (0.021)	1.186	7.411
QH	H2 (3)	0	0	0	16	0.708 (0.091)	0.338	2.101
SC1	H2 (3), H5 (7)	0.467 (0.132)	0.046	0.933	12	0.646 (0.101)	0.316	1.972
SC2	H5 (3), H6 (12)	0.343 (0.128)	0.017	0.343	12	0.607 (0.106)	0.855	5.326
YN	H6 (15)	0	0	0	9	0.515 (0.111)	0.127	0.795
GZ	H4 (15)	0	0	0	13	0.745 (0.083)	0.219	1.368
Mean		0.662 (0.033)	0.040	0.820		0.647 (0.032)	0.543	3.012

**TABLE 3:** Analysis of molecular variance of the chloroplast haplotypes and internal transcribed spacer (ITS) sequence types for the populations of *Gentiana crassicaulis*

Source of variation	Chloroplast DNA					ITS				
	*df*	SS	VC	PV (%)	*Fst*	*df*	SS	VC	PV (%)	*Fst*
Among populations	10	39.312	0.38481	85.61		10	58.158	0.14817	9.75	
Within populations	102	6.600	0.06471	14.39		319	437.267	1.37074	90.25	
Total	112	45.912	0.44951		0.8561*	329	495.424	1.51891		0.0975*

Abbreviations: *df*, degrees of freedom; *Fst*, fixation index; PV, percentage of variation; SS, sum of squares; VC, variance component.

*p < .001.

The haplotype diversity (*Hd*) of ITS per population ranged from 0.515 to 0.968, with an average value of 0.647, and the π value ranged from 0.117 to 1.186, with an average value of 0.543. GS population had the most abundant haplotypes and the highest *Hd* and π values, showing the highest diversity, followed by XZ1 population. YN population had the lowest diversity (Table 2). The *Ht* of ITS and the *Hs* were 0.794 and 0.648, respectively. The *Fst* was high (0.09755), and the *Nm* was extensive (2.33). *Nst* (0.145) was smaller than *Gst* (0.183), indicating no phylogenetic structure. AMOVA based on ITS suggested high‐level within‐population differentiation (90.25% of the total diversity) and low‐level among‐population differentiation (9.75%) (Table 3). In terms of haplotypes, there were abundant haplotypes within each population. Although the shared haplotypes of S1 and S2 accounted for a large proportion of the haplotypes among the populations, there was only one variable site between S1 and S2. The results of the variation analysis of the ITS sequences significantly differed from the results based on the chloroplast sequences.

### 3.5 Demographic analyses

**FIGURE 4:**
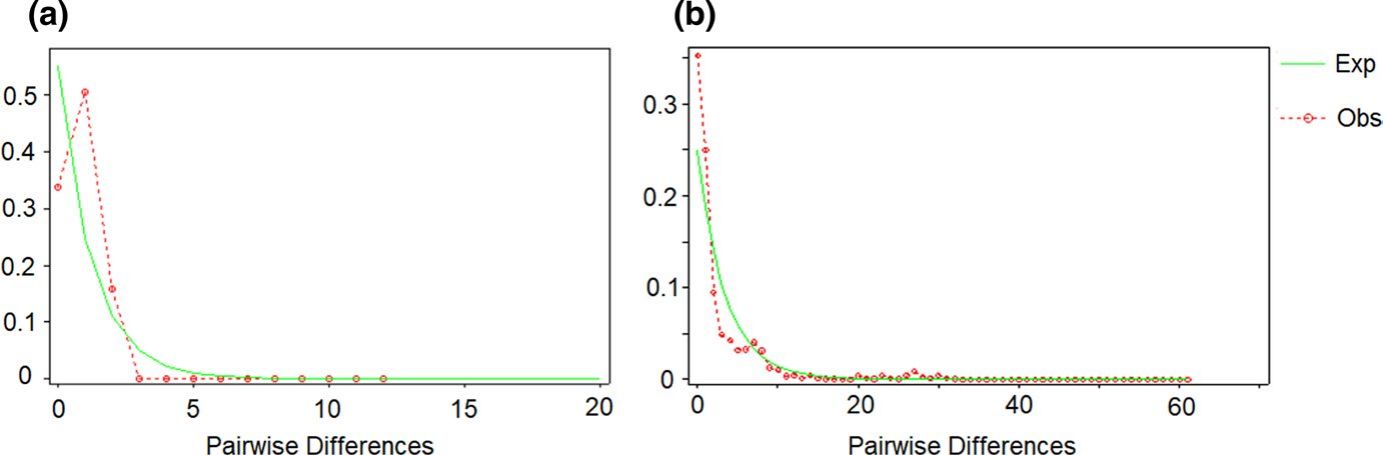
Mismatch distribution analyses of the chloroplast (a) and internal transcribed spacer (b) haplotypes for the *Gentiana crassicaulis* populations. The solid and dotted lines represent the expected and observed distributions, respectively

Based on the chloroplast data, the mismatch distribution of *G. crassicaulis* showed a single‐peak curve (Figure 4a), and the neutral test showed that the Tajima's D value and Fu's Fs value of *G. crassicaulis* were 0.78742 and 0.961 (*p* > .10), respectively, indicating that the populations may have not experienced rapid expansion events (Fu & Li, 1993; Tajima, 1989). Based on the ITS data, the expected distribution curve was found to be overall consistent with the observed distribution curve, and a graph of non‐unimodal distribution was obtained (Figure 4b), indicating that the populations may have not experienced expansion events (Schneider & Excoffier, 1999).

### 3.6 Estimation of divergence time

**FIGURE 5:**
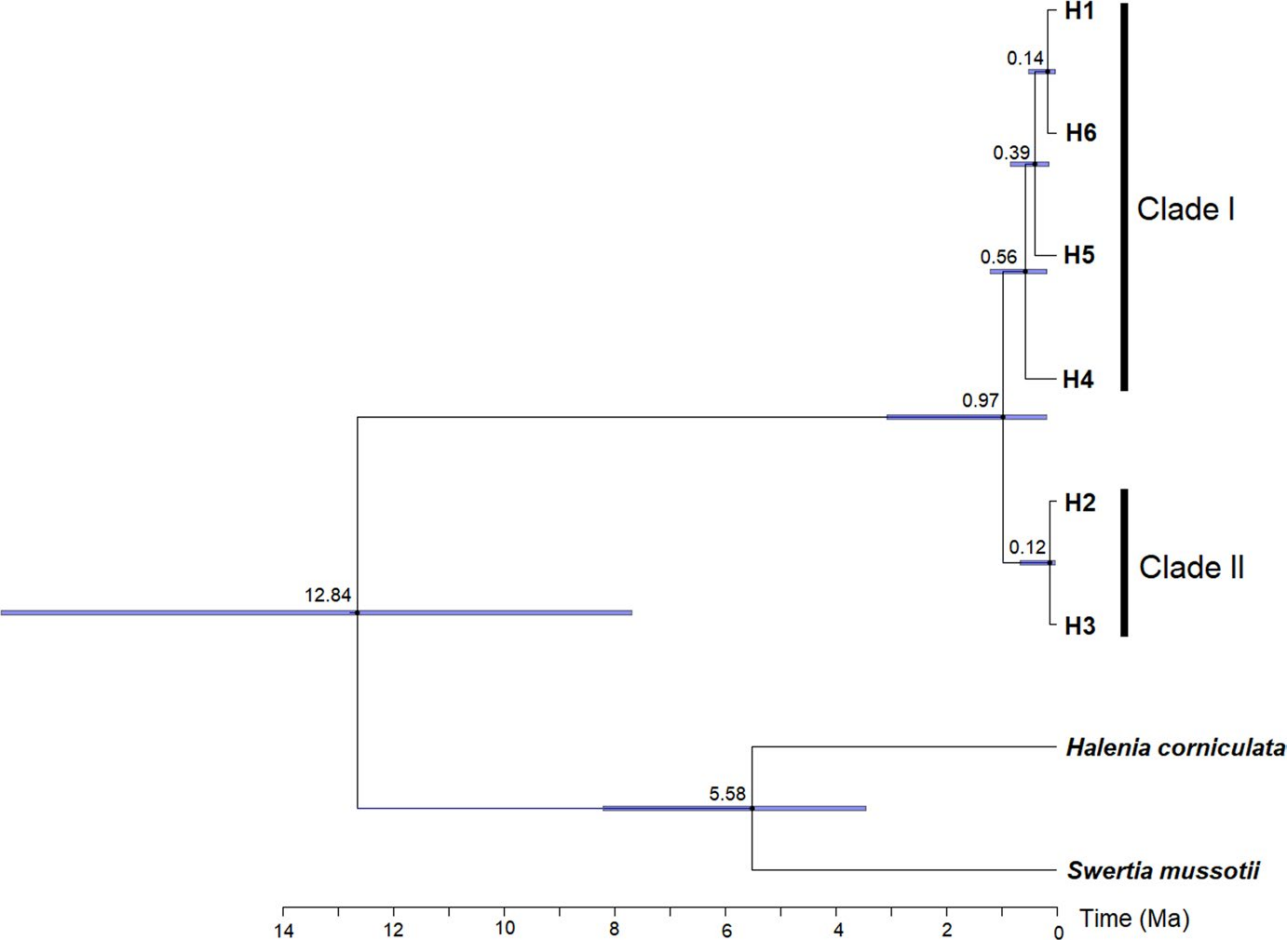
Divergence times of the detected chloroplast haplotypes in *G. crassicaulis* and two outgroups estimated using BEAST. Numbers on the branch indicate the estimated divergence time age of the node (Ma), with blue bars indicating the 95% highest posterior density intervals

BEAST was used to analyze the divergence time of the chloroplast haplotypes of *G. crassicaulis*, with *Swertia Mussotii* and *Halenia Corniculata* as outgroups (Figure 5). Six haplotypes (H1–H6) formed a monophyletic group (posterior probability = 1), which was clustered into two clades (Clade I and Clade II). H1, H4, H5, and H6 formed Clade I, and H2 and H3 formed Clade II. The divergence time between Clade I and Clade II occurred at 0.97 Ma (95% HPD, 3.39–0.13). For Clade I, the diversification started at 0.56 Ma (95% HPD, 1.24–0.16), and the latest diversification of H1 and H6 occurred at 0.14 Ma (95% HPD, 0.29–0.018). The divergence time of the two haplotypes in Clade II occurred at 0.12 Ma (95% HPD, 0.58–0.021).

### 3.7 Ecological niche models

**FIGURE 6:**
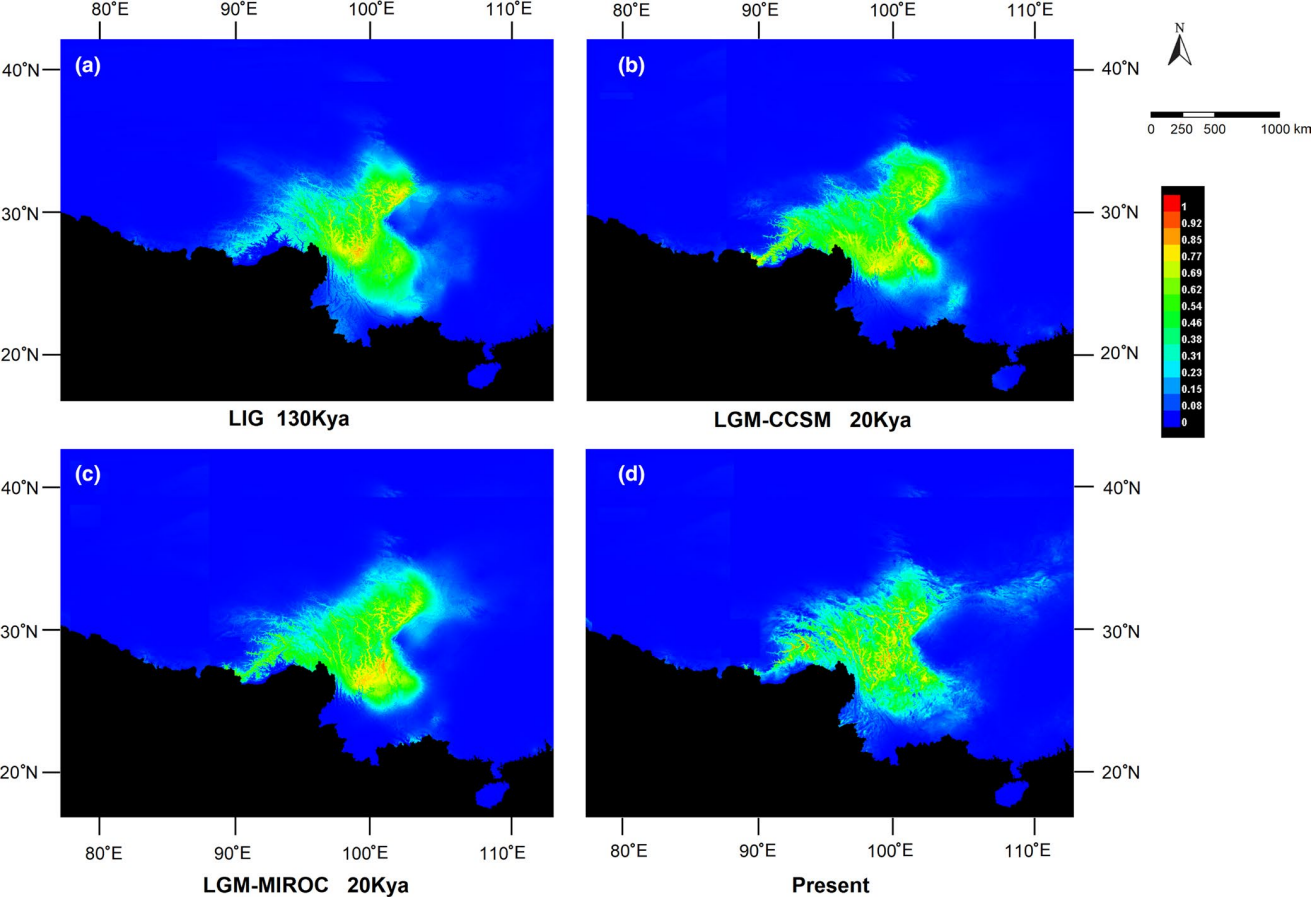
Predicted distribution of *Gentiana crassicaulis* according to ecological niche modeling by using MAXENT 3.4.0. Predicted distribution during the (a) LIG period, (b) LGM period based on CCSM climate simulation, and (c) LGM period based on the model for MIROC climate simulation. (d) The present predicted distribution

The traditional AUC and TSS methods of estimation of model accuracy of all the models in different periods indicated good predictability for the models. The AUC value of the model in each period ranged between 0.974 and 0.980, and TSS value ranged between 0.765 and 0.793 (Table S2), indicating high stability and accuracy. The predicted distribution of the LIG and LGM (CCSM, MIROC) periods and the present is shown in Figure 6. The distribution of *G. crassicaulis* in the QTP showed no significant change during the LGM period compared with the distribution during the LIG period, except for a slight contraction in the southeast region. Focusing on the distribution area with a high probability (>0.8), the two models of the LGM period both showed a significant southward shift compared with the models of the LIG period, which were mainly concentrated in Yunnan and Guizhou, and the distribution area with a high probability at the present spread throughout.

## 4 DISCUSSION

In this study, the species diversity and phylogeographic pattern of *G. crassicaulis* were studied by using two genomic fragments, namely maternal chloroplast sequences and parental nrITS sequences. Chloroplasts have their own complete set of genomes (McFadden, 2001), which have become a powerful tool for phylogenetic research of plants (Moore et al., 2007; Ni et al., 2015; Nock et al., 2011). nrITS is a transcription interval region with a fast mutation rate dictated by ribosomal DNA and is inherited by both parents. ITS is an important molecular marker for the systematic evolution of plants and has been widely used in the study of systematic classification and evolution of angiosperms (Eickbush & Eickbush, 2007; Rosselló et al., 2006). ITS contains highly repetitive tandem sequences, and the repeating units can be homozygous or nearly homozygous among most species due to concerted evolution (Xiao et al., 2010). However, some species have the phenomenon of incomplete concerted evolution, leading to the possibility of high diversity within species and even within the genome of the same individual (Bailey et al., 2003; Xiao et al., 2010). Incomplete concerted evolution leads to a low degree of homozygosity among rDNA repeats. Thus, results from direct sequencing of PCR products may be uninformative, and only one cloning sequence obviously cannot reflect the variation among the repeats (Li & Yang, 2002). Therefore, the polymorphism of the ITS region can only be evaluated using a large number of cloning sequences. There are also many studies that have discovered evolutionary process by analyzing the concerted evolution of the ITS region (Ochieng et al., 2007). Based on previous studies (Ni et al., 2016; Xiong et al., 2015), 330 cloning sequences of the ITS region of *G. crassicaulis* were analyzed here. The concerted evolution of *G. crassicaulis* is still ongoing. According to the distribution of the haplotypes, all the populations could be divided into two main clades, one including the Yunnan and Guizhou populations, and the other including the populations from the other regions.

Chloroplast DNA analysis showed that the gene flow among the populations of *G. crassicaulis* was small, and the average genetic diversity within the populations was much lower than the total genetic diversity, with most of the genetic variation existing among the populations (85.61%). However, the analysis of the ITS haplotypes showed that the gene flow among the populations was extensive, and most of the genetic variation existed within the populations (90.25%). This variation was found to be much higher than the genetic variation among the populations. Both ITS and chloroplast sequences are commonly used in phylogeographic studies, and the results in most species are basically consistent (Christe et al., 2014; Fu et al., 2016; Yang et al., 2018). However, there are also studies indicating that chloroplast and ITS markers can have different phylogeographic patterns within the same species (Gao et al., 2016). In this study, the analyses of the chloroplast and ITS sequences yielded quite different results. Due to the geographical environment of *G. crassicaulis*, seed migration and gene flow among the seeds are limited. Consequently, many chloroplast haplotypes are confined to a single population, except for H1, which is shared by many populations in Tibet, and there is a great chloroplast DNA variation among the populations. Additionally, the restriction of pollen transfer after population differentiation is relatively less than that of seed, and thus an extensive gene flow can ensue, subsequently resulting in a low degree of genetic variation in ITS sequence among the populations.

In our previous study, amplified fragment length polymorphism markers were used to analyze the genetic diversity of 163 individuals from 20 populations of *G. crassicaulis* (Zong et al., 2020). The results showed that the genetic variation mainly existed among the populations (87%), and the genetic variation within the populations was small (13%), close to the results of the chloroplast DNA analysis in the presented study. Phylogenic analysis in the previous study showed that the populations could be divided into two clades, with one including the Tibet, Gansu, Qinghai, and Sichuan populations, and the other including the Guizhou and Yunnan populations, consistent with the results of the ITS analysis in the presented study. Principal component analysis (PCA) and the Mantel test showed that the genetic distance among the populations significantly increased with the increase in the geographical distance. Geographical distribution may be one of the important factors affecting the genetic structure of *G. crassicaulis*.

There is a distinct differentiation, usually accompanied by a unique phylogeographic structure, among the populations of *G. crassicaulis*. However, no obvious phylogeographic structure was detected based on the chloroplast and ITS data, and the contradiction between the obvious genetic differentiation and indistinct phylogeographic structure might be caused by the large number of fixed private chloroplast haplotypes and the wide distribution of the main ITS haplotypes.

The chloroplast analysis revealed that the populations with high diversity were mainly from the edge of the QTP platform, whereas the populations on the platform were fixed with a single haplotype. In the ITS analysis, the GS population located at the edge of the platform was found to be the population with the highest diversity. These results suggest that the diversification may have occurred at the edge of the platform, and the species may have spread from the edge to the platform. Furthermore, the dating analysis based on the DNA data suggested that the diversity of *G. crassicaulis* at the edge of the QTP platform occurred before the LGM period. Therefore, the species may have undergone diversification at the edge of the QTP platform and completed its expansion from the edge to the platform.

Species in the northeast of QTP experienced an expansion to the plateau after the last glacial period, and the main branches of the species in the southeast of QTP were differentiated at the beginning of the Quaternary due to the uplift of plateau (Yu & Zhang, 2013). During the Quaternary glacial period, there were many refugia (including microrefugia) on the platform and at the edge of QTP, and only small expansion or vertical movement occurred in the valley during the interglacial and postglacial period. However, some species, such as *Tsuga Dumosa* (Cun & Wang, 2010) and *Pedicularis longiflora* (Yang et al., 2008), underwent large‐scale expansion, most of which occurred before the last glacial period, with last glacial period having little effect on their distribution pattern. Some cold‐tolerant and drought‐tolerant plants, such as *Potentilla fruticosa* (Sun et al., 2010) and *Reaumuria soongarica* (Li et al., 2012), were completely different from most species in their response to the glacial period, and usually expanded during the glacial period. ENM reconstruction showed that the distribution of *G. crassicaulis* during the LGM period was slightly limited, and the range of variation before and during LGM period was not large. The distribution area with a high probability during the LGM transferred from the plateau platform to the southern edge of the plateau. This situation indicated that LGM did not reduce the distribution area of *G. crassicaulis* but promoted the further colonization at the edge of the plateau, contrary to what was predicted for some other species (Meng et al., 2007; Yang et al., 2008). The colonization led to an increase in haplotype diversity in the populations at the edge of the plateau, similar to that of *Metagentiana striata* (Chen et al., 2008). The ENM reconstruction also showed that the distribution with a high probability shrank at the southern edge of the plateau with the increase in temperature after the LGM period, while the probability of the distribution across the platform increased significantly. This continued expansion during the glacial period supports the hypothesis that no large ice sheets formed on the QTP during the Pleistocene glacial period, and periodic glaciation–interglacial periods also affected the distribution of alpine plants. Mismatch analysis and neutral test showed that *G. crassicaulis* did not experience sudden expansion recently on the QTP, and the population remained relatively stable. Phylogenetic analysis and estimation of divergence time indicated that the species originated late and experienced a short period of differentiation among haplotypes. In the late Miocene, a series of mountains in southwest China continued to rise, such as the Himalayas and the Hengduan Mountains, which blocked the monsoon from the Indian Ocean and made the climate cooler and drier. Some woody plants migrated southward or became extinct, and herbaceous plants flourished more (Quade et al., 1989). *Gentian* should have undergone radiative differentiation during this period, giving rise to many new species.

Several phylogeographic models have been reported to explain the distribution patterns of the alpine plants in the QTP. The phylogeography of *G. crassicaulis* indicates that the species survived at the edge of the QTP during the LGM, and there was a tendency to migrate from the platform to the southern edge and extend to the platform after the LGM. This model is similar to that of *G. straminea* but different from that of common alpine plants and may provide a reference for further research on the responses of alpine species to the Quaternary climate change.

## CONFLICT OF INTEREST

The authors declare that they have no conflict of interest.

## AUTHOR CONTRIBUTIONS

**Lianghong Ni:** Conceptualization (equal); Data curation (lead); Formal analysis (lead); Funding acquisition (equal); Investigation (equal); Methodology (equal); Resources (equal); Software (lead); Validation (lead); Writing – original draft (lead). **Weitao Li:** Data curation (equal); Formal analysis (equal); Investigation (equal); Methodology (equal); Software (lead); Validation (equal); Writing – original draft (equal). **Zhili Zhao:** Conceptualization (lead); Data curation (equal); Funding acquisition (lead); Investigation (lead); Methodology (lead); Project administration (lead); Resources (lead); Software (equal); Supervision (lead); Validation (lead); Writing – original draft (equal); Writing – review & editing (lead). **Dorje Gaawe:** Conceptualization (equal); Investigation (equal); Resources (equal); Supervision (equal). **Tonghua Liu:** Funding acquisition (equal); Project administration (equal); Resources (equal).

## Supporting information

Fig S1

Fig S2

Table S1

Table S2

Table S3

Table S4

Table S5

Table S6

## Data Availability

GenBank accessions: sequences of chloroplast genomes (KY595457–KY595463, KY606171); sequences of chloroplast fragments (MW112247–MW112698); and sequences of ITS clones (MF506888–MF506967, MF785125–MF785294, and MF981182–MF981261). Genotyping data are available on Dryad at https://doi.org/10.5061/dryad.z8w9ghxf1.
