# Supplementary figures and images for: Migration patterns of Gentiana crassicaulis, an alpine gentian endemic to the Himalaya–Hengduan Mountains
Source: Ecol Evol. 2022 Mar 18;12(3):e8703. doi: 10.1002/ece3.8703 (PMC8933255; doi:10.1002/ece3.8703)

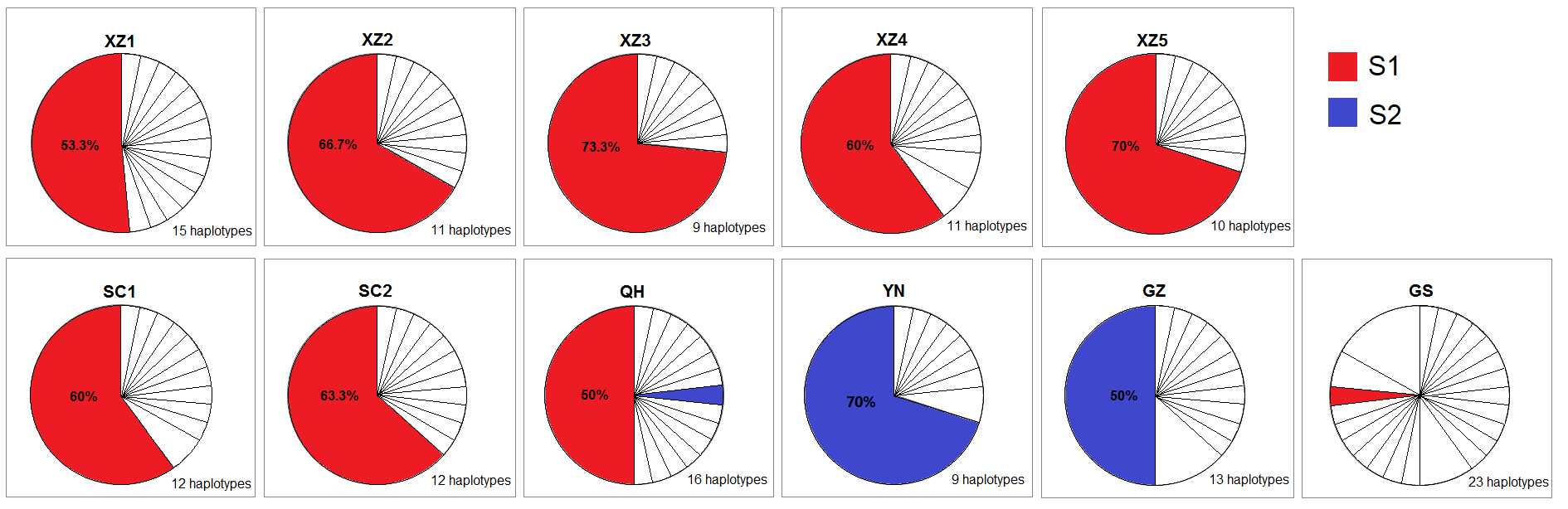


**FIGURE S1** ITS haplotypes of each population.

Supplement: Supplementary file 1 — Fig S1 [file ECE3-12-e8703-s002.docx]

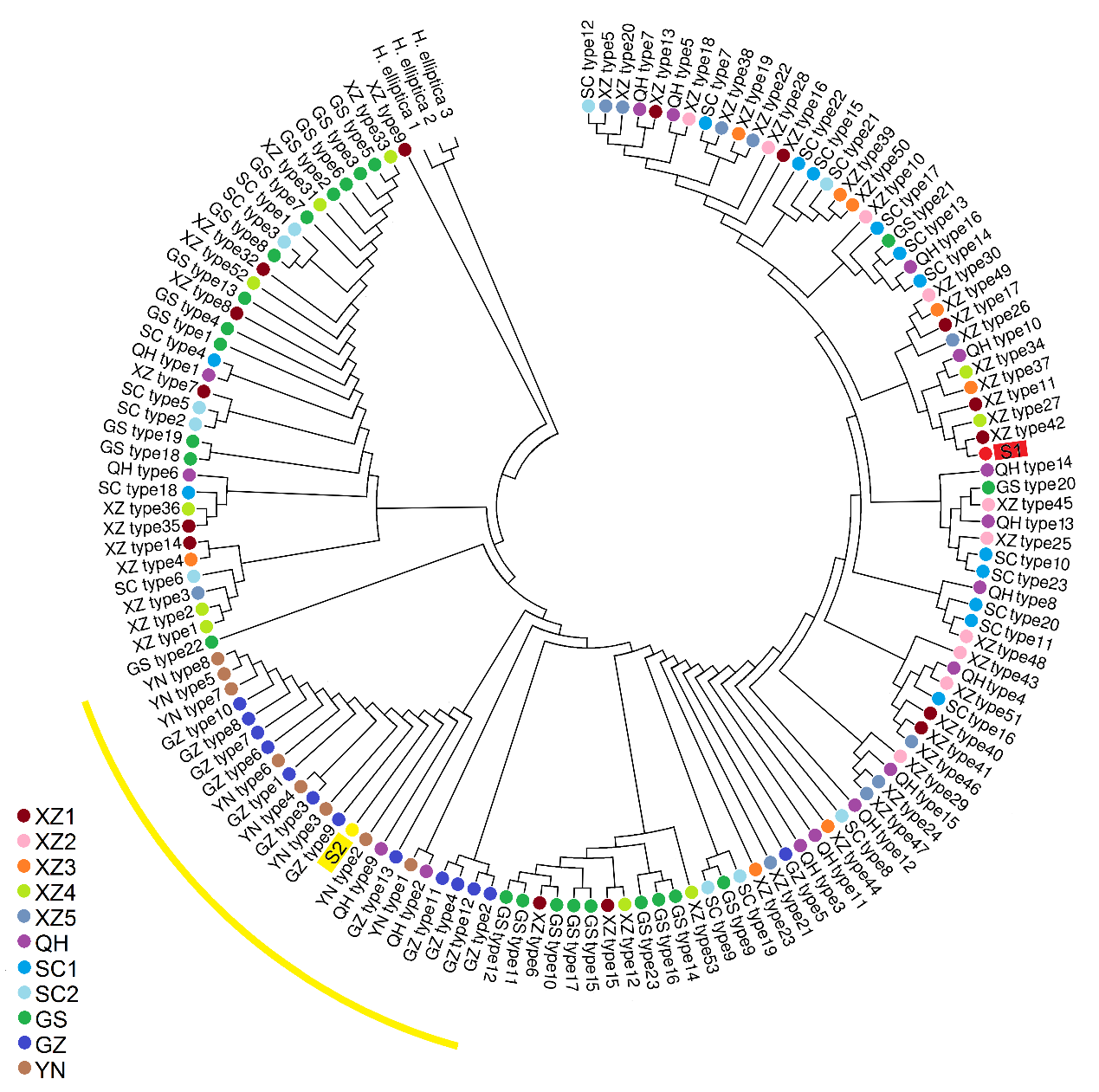


**FIGURE S2** MP phylogenetic tree based on ITS haplotypes, with *Halenia elliptica* as an outgroup.

Supplement: Supplementary file 2 — Fig S2 [file ECE3-12-e8703-s003.docx]
