# Supplementary material for: Migration patterns of Gentiana crassicaulis, an alpine gentian endemic to the Himalaya–Hengduan Mountains
Source: Ecol Evol. 2022 Mar 18;12(3):e8703. doi: 10.1002/ece3.8703 (PMC8933255; doi:10.1002/ece3.8703)
Supplement: Supplementary file 3 — Table S1 [file ECE3-12-e8703-s007.docx]

**TABLE S1** Sample information

| Voucher | Indiv. No. | Code | Locality | GPS coordinate (North; East) | Alt.(m) |
| --- | --- | --- | --- | --- | --- |
| 2016XZ011^*^ | 15 | XZ1 | Changdu, Tibet | N 31°09.173ʹ; E 97°14.025ʹ | 4027 |
| XZ201214^*^ | 6 | XZ2 | Dingqing, Tibet | N 31°21.458ʹ; E 95°52.533ʹ | 4561 |
| 2016XZ009 | 3 | XZ3 | Yelashan, Tibet | N 30°08.997ʹ; E 97°17.618ʹ | 4543 |
| 2017XZ006 | 10 | XZ4 | Bomi, Tibet | N 29°47.940ʹ; E 95°49.381ʹ | 2838 |
| 2017XZ008 | 6 | XZ5 | Zuogong, Tibet | N 29°40.487ʹ; E 97°49.332ʹ | 3839 |
| GS201601^*^ | 15 | GS | Maqu, Gansu | N 34°03.815ʹ; E 102°04.138ʹ | 3573 |
| QH201405^*^ | 3 | QH | Banma, Qinghai | N 32°46.608ʹ; E 100°49.695ʹ | 3402 |
| SC201601^*^ | 10 | SC1 | Kangding, Sichuan | N 29°54.618ʹ; E 101°59.538ʹ | 3835 |
| SC201602^*^ | 15 | SC2 | Daofu, Sichuan | N 30°29.862ʹ; E 101°29.727ʹ | 3461 |
| YN201601^*^ | 15 | YN | Lijiang, Yunnan | N 27°00.938ʹ; E 100°09.601ʹ | 3298 |
| GZ201601^*^ | 15 | GZ | Weining, Guizhou | N 27°06.396ʹ; E 104°07.377 ʹ | 2552 |

^*^Chloroplast genome sequencing.
