# Supplementary material for: Migration patterns of Gentiana crassicaulis, an alpine gentian endemic to the Himalaya–Hengduan Mountains
Source: Ecol Evol. 2022 Mar 18;12(3):e8703. doi: 10.1002/ece3.8703 (PMC8933255; doi:10.1002/ece3.8703)

**TABLE S2** Area under curve (AUC) and true skill statistic (TSS) values of MaxEnt models in different periods

| period | AUC | TSS |
| --- | --- | --- |
| LIG | 0.976 | 0.781 |
| LGM-CCSM | 0.974 | 0.765 |
| LGM-MIROC | 0.977 | 0.793 |
| Present | 0.980 | 0.788 |


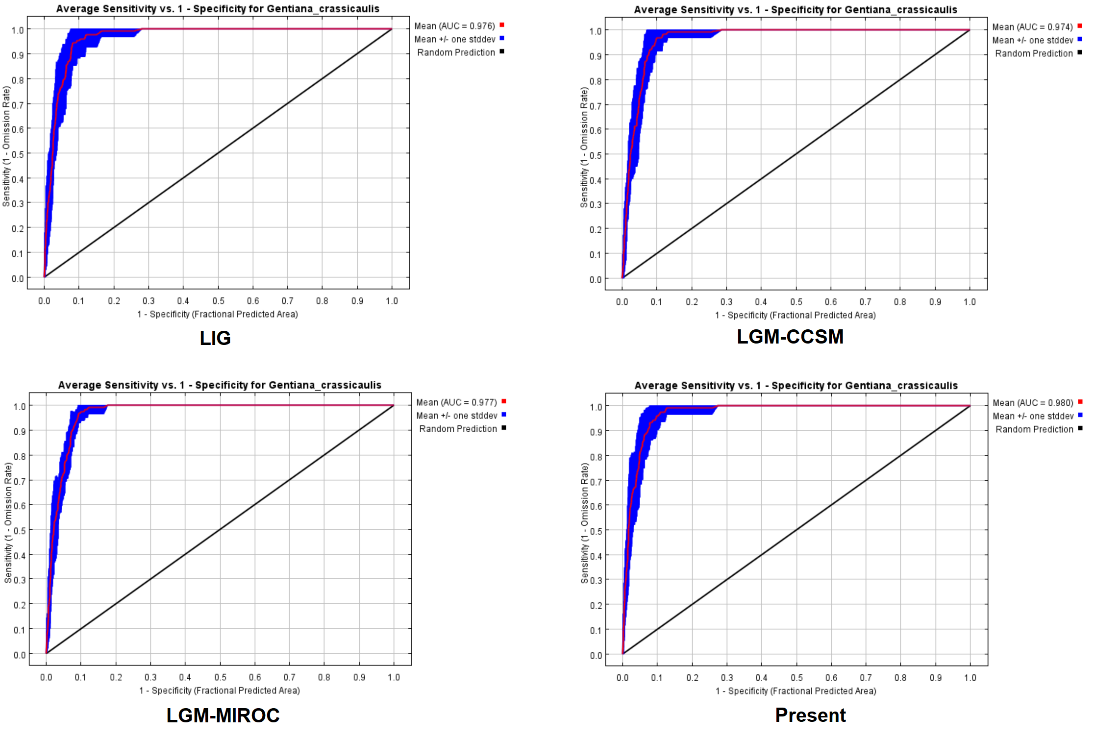

Supplement: Supplementary file 4 — Table S2 [file ECE3-12-e8703-s004.docx]
