# Supplementary material for: Migration patterns of Gentiana crassicaulis, an alpine gentian endemic to the Himalaya–Hengduan Mountains
Source: Ecol Evol. 2022 Mar 18;12(3):e8703. doi: 10.1002/ece3.8703 (PMC8933255; doi:10.1002/ece3.8703)
Supplement: Supplementary file 6 — Table S4 [file ECE3-12-e8703-s005.docx]

**TABLE S4** Selection of major climate factors used in the study and their contribution rate in different periods

| Code | Bioclimatic factor variable | Contribution rate (%) | | | |
| --- | --- | --- | --- | --- | --- |
|  |  | LIG | LGM- CCSM | LGM- MIROC | Current |
| bio1* | Annual mean temperature | 5.9 | 12.8 | 5.6 | 11.8 |
| bio2* | Mean diurnal range (mean of monthly (max temp-min temp) ) | 0.5 | 2.1 | 2.7 | 0.8 |
| bio3* | Isothermality (bio2 /bio7) (×100) | 33.8 | 43 | 34.4 | 43.1 |
| bio4 | Temperature seasonality (standard deviation×100) |  |  |  |  |
| bio5 | Max temperature of warmest month |  |  |  |  |
| bio6 | Min temperature of coldest month |  |  |  |  |
| bio7* | Temperature annual range (bio5-bio6) | 41.7 | 4.2 | 5.7 | 9.5 |
| bio8 | Mean temperature of wettest quarter |  |  |  |  |
| bio9 | Mean temperature of driest quarter |  |  |  |  |
| bio10 | Mean temperature of warmest quarter |  |  |  |  |
| bio11 | Mean temperature of coldest quarter |  |  |  |  |
| bio12* | Annual precipitation | 2 | 32.9 | 33.5 | 33.1 |
| bio13 | Precipitation of wettest month |  |  |  |  |
| bio14 | Precipitation of driest month |  |  |  |  |
| bio15* | Precipitation seasonality (coefficient of variation) | 0.8 | 0.5 | 2.7 | 0.9 |
| bio16 | Precipitation of wettest quarter |  |  |  |  |
| bio17* | Precipitation of driest quarter | 14.8 | 4.4 | 15.5 | 0.6 |
| bio18* | Precipitation of warmest quarter | 0.5 | 0.1 | 0.1 | 0.1 |
| bio19 | Precipitation of coldest quarter |  |  |  |  |

Note: * The variable is the selected climate factor．
