# Supplementary material for: Migration patterns of Gentiana crassicaulis, an alpine gentian endemic to the Himalaya–Hengduan Mountains
Source: Ecol Evol. 2022 Mar 18;12(3):e8703. doi: 10.1002/ece3.8703 (PMC8933255; doi:10.1002/ece3.8703)
Supplement: Supplementary file 7 — Table S5 [file ECE3-12-e8703-s008.docx]

|  | XZ1 | XZ2 | GS | GZ | SC1 | SC2 | QH | YN |
| --- | --- | --- | --- | --- | --- | --- | --- | --- |
| GenBank ID | KY595457 | KY595458 | KY595459 | KY595460 | KY595461 | KY595462 | KY606171 | KY595463 |
| Total (bp) | 148757 | 148757 | 148788 | 148777 | 148778 | 148755 | 148724 | 148754 |
| LSC region (bp) | 81143 | 81143 | 81174 | 81162 | 81164 | 81141 | 81110 | 81140 |
| SSC region (bp) | 17070 | 17070 | 17070 | 17071 | 17070 | 17070 | 17070 | 17070 |
| IR region (bp) | 25272 | 25272 | 25272 | 25272 | 25272 | 25272 | 25272 | 25272 |
| AT content (%) | 62.28 | 62.28 | 62.29 | 62.29 | 62.29 | 62.28 | 62.28 | 62.28 |
| GC content(%) | 37.72 | 37.72 | 37.71 | 37.71 | 37.71 | 37.72 | 37.72 | 37.72 |

**TABLE S5-1** Base composition of *Gentiana* *crassicaulis* chloroplast genomes

**TABLE S5-2** SNPs among *G. crassicaulis* chloroplast genomes

| Nucleotide position according to YN | Location | Region | XZ1 | XZ2 | GS | GZ | SC1 | SC2 | QH | YN |
| --- | --- | --- | --- | --- | --- | --- | --- | --- | --- | --- |
| 2553 | *matK** | LSC | G | G | C | C | C | C | C | G |
| 4116 | *trnK-UUU* intron | LSC | C | C | T | C | C | C | C | C |
| 9513 | *atpA** | LSC | G | G | G | A | G | G | G | G |
| 10593 | *atpF* intron | LSC | T | T | T | T | T | T | T | A |
| 12639 | *atpH-atpI* | LSC | T | T | T | T | T | T | T | C |
| 16595 | *rpoC2** | LSC | C | C | T | C | T | C | C | C |
| 26400 | *rpoB-trnC-GCA* | LSC | C | C | C | C | C | C | C | T |
| 26798 | *trnC-GCA-petN* | LSC | C | C | C | T | C | C | C | C |
| 30448 | *trnT-GGU-psbD* | LSC | C | C | C | C | C | C | C | A |
| 32520 | *psbC** | LSC | C | C | C | C | C | C | C | T |
| 32901 | *psbC** | LSC | C | C | C | T | C | C | C | C |
| 44119 | *trnS-GGA-rps4* | LSC | T | T | T | T | T | T | T | G |
| 44221 | *rps4** | LSC | C | C | C | C | C | C | C | T |
| 50863 | *atpE** | LSC | G | G | G | G | G | G | G | A |
| 52748 | *atpB-rbcL* | LSC | T | T | T | T | T | T | T | G |
| 56623 | *accD** | LSC | G | G | G | C | G | G | G | G |
| 57639 | *ycf4** | LSC | C | C | C | T | C | C | C | C |
| 58261 | *ycf4-cemA* | LSC | T | T | T | C | T | T | T | T |
| 60624 | *petA-psbJ* | LSC | C | C | C | C | C | A | C | C |
| 60678 | *petA-psbJ* | LSC | G | G | G | A | G | G | G | G |
| 63718 | *petG-trnW-CCA* | LSC | A | A | A | C | A | A | A | A |
| 66956 | *rpl20-rps12* | LSC | A | A | A | A | A | A | A | G |
| 67424 | *rps12-clpP* | LSC | T | T | A | A | A | A | A | A |
| 71977 | *psbN** | LSC | A | A | A | A | G | A | A | A |
| 72572 | *petB* intron | LSC | G | G | G | G | G | G | G | A |
| 75494 | *petD-rpoA* | LSC | G | G | G | G | G | A | G | G |
| 78488 | *rpl14-rpl16* | LSC | G | G | G | A | G | G | G | G |
| 79075 | *rpl16* intron | LSC | G | G | A | G | A | G | A | G |
| 93653 | *ndhB-rps7* | IR | C | C | C | C | C | C | A | C |
| 104254 | *rrn5-trnR-ACG* | IR | C | C | C | A | C | C | C | C |
| 111140 | *ccsA** | SSC | T | T | T | T | T | G | T | T |
| 111165 | *ccsA-ndhD* | SSC | C | C | C | C | C | C | C | T |
| 119740 | *ycf1** | SSC | G | G | G | G | G | C | G | G |
| 119900 | *ycf1** | SSC | G | G | G | G | G | G | G | C |
| 120123 | *ycf1** | SSC | A | A | C | A | A | A | A | A |
| 120801 | *ycf1** | SSC | G | G | A | A | A | A | A | A |
| 122310 | *ycf1** | SSC | T | T | G | G | G | G | G | G |

*The asterisks indicate SNPs located in [protein](javascript:;) [coding](javascript:;) [gene](javascript:;)s.

**TABLE S5-3** Difference in SSR among *G. crassicaulis* chloroplast genomes

| Nucleotide position according to YN | Location | Region | XZ1 | XZ2 | GS | GZ | SC1 | SC2 | QH | YN |
| --- | --- | --- | --- | --- | --- | --- | --- | --- | --- | --- |
| 6698 | *psbI-trnS-GCU* | LSC | 10A | 10A | 10A | 11A | 10A | 9A | 10A | 10A |
| 6962 | *trnS-GCU-trnG-UCC* | LSC | 12A | 12A | 11A | 11A | 11A | 12A | 11A | 13A |
| 26345 | *rpoB-trnC-GCA* | LSC | 11A | 11A | 9A | 8A | 9A | 10A | 9A | 10A |
| 28915 | *psbM-trnD-GUC* | LSC | 13T | 13T | 13T | 13T | 13T | 13T | 13T | 12T |
| 30278 | *trnT-GGU-psbD* | LSC | 8A | 8A | 8A | 8A | 8A | 8A | 8A | 7A |
| 43073 | *ycf3* intron1 | LSC | 10A | 10A | 11A | 11A | 10A | 11A | 10A | 9A |
| 45333 | *trnT-UGU-trnL-UAA* | LSC | 10A | 10A | 10A | 9A | 10A | 10A | 10A | 9A |
| 47285 | *trnF-GAA-ndhJ* | LSC | 2T | 2T | 3T | 2T | 3T | 2T | 3T | 2T |
| 75308 | *petD-rpoA* | LSC | 10A | 10A | 9A | 10A | 9A | 10A | 9A | 10A |
| 75468 | *petD-rpoA* | LSC | 9A | 9A | 9A | 9A | 9A | 10A | 9A | 9A |
| 116389 | *ndhA intron* | SSC | 8T | 8T | 8T | 9T | 8T | 8T | 8T | 8T |

**Table S5-4** InDels among *G. crassicaulis* chloroplast genomes

| Nucleotide position according to YN | Location | Region | sequence | XZ1 | XZ2 | GS | GZ | SC1 | SC2 | QH | YN |
| --- | --- | --- | --- | --- | --- | --- | --- | --- | --- | --- | --- |
| 150 | *trnH-GUG-psbA* | LSC | CTTTTTTTAACTCT  TTTTTATATTTT* | - | - | + | + | + | - | + | - |
| 5113 | *trnK-UUU-Ψrps16* | LSC | TATAAC* | - | - | + | + | + | + | + | + |
| 26636 | *trnC-GCA-petN* | LSC | TTTTTCAA* | + | + | - | - | - | - | - | - |
| 52636 | *atpB-rbcL* | LSC | GCTATTTAGAT* | + | + | + | + | + | + | + | - |
| 59464 | *cemA-petA* | LSC | TTATTCCAGTAAAA* | - | - | - | - | - | - | - | + |
| 60558 | *petA-psbJ* | LSC | GTTCGTAAAAAGAACCAAATTCTTCTTGGTAATTATGTATGATCAAAGATCAAA | + | + | + | + | + | + | - | + |
| 65367 | *rpl33-rps18* | LSC | TAAGGGAAG* | + | + | ++ | - | + | + | + | + |
| 68882 | *clpp* intron1 | LSC | TCTTATA* | - | - | - | + | - | - | - | - |

*The asterisks indicate units of repeats.
