# Supplementary material for: Migration patterns of Gentiana crassicaulis, an alpine gentian endemic to the Himalaya–Hengduan Mountains
Source: Ecol Evol. 2022 Mar 18;12(3):e8703. doi: 10.1002/ece3.8703 (PMC8933255; doi:10.1002/ece3.8703)
Supplement: Supplementary file 8 — Table S6 [file ECE3-12-e8703-s001.docx]

**TABLE S6-1** GenBank ID of ITS cloning sequences

| Voucher specimens | GenBank ID  ((*n* = 10)) | Voucher specimens | GenBank ID  (*n* = 10) | | Voucher specimens | GenBank ID  (*n* = 10) | |
| --- | --- | --- | --- | --- | --- | --- | --- |
| XZ201214-1 | MF506888- MF506897 | XZ201214-2 | | MF785235- MF785244 | XZ201214-3 | | MF785245- MF785254 |
| 2016XZ009-1 | MF506898- MF506907 | 2016XZ009-2 | | MF981182- MF981191 | 2016XZ009-3 | | MF981192- MF981201 |
| 2016XZ011-1 | MF785125- MF785134 | 2016XZ011-2 | | MF785135- MF785144 | 2016XZ011-3 | | MF785145- MF785154 |
| 2017XZ006-1 | MF981202- MF981211 | 2017XZ006-2 | | MF981212- MF981221 | 2017XZ006-3 | | MF981222- MF981231 |
| 2017XZ008-1 | MF981232- MF981241 | 2017XZ008-2 | | MF981242- MF981251 | 2017XZ008-3 | | MF981252- MF981261 |
| GS201601-1 | MF506908- MF506917 | GS201601-2 | | MF785155- MF785164 | GS201601-3 | | MF785165- MF785174 |
| QH201405-1 | MF506958- MF506967 | QH201405-2 | | MF785255- MF785264 | QH201405-3 | | MF785265- MF785274 |
| SC201601-1 | MF506928- MF506937 | SC201601-2 | | MF785195- MF785204 | SC201601-3 | | MF785205- MF785214 |
| SC201602-1 | MF506938- MF506947 | SC201602-2 | | MF785215- MF785224 | SC201602-3 | | MF785225- MF785234 |
| YN201601-1 | MF506948- MF506957 | YN201601-2 | | MF785275- MF785284 | YN201601-3 | | MF785285- MF785294 |
| GZ201601-1 | MF506918- MF506927 | GZ201601-2 | | MF785175- MF785184 | GZ201601-3 | | MF785185- MF785194 |

**TABLE S6-2** GenBank ID of chloroplast sequences

| Voucher  specimens | n | *rpl16* intron | *rpl33* | *rpl33-rps18* | *trnC-GCA-petN* |
| --- | --- | --- | --- | --- | --- |
| XZ201214 | 6 | MW112247- MW112252 | MW112360- MW112365 | MW112473- MW112478 | MW112586- MW112591 |
| 2016XZ009 | 3 | MW112253- MW112255 | MW112366- MW112368 | MW112479- MW112481 | MW112592- MW112594 |
| 2016XZ011 | 15 | MW112256- MW112270 | MW112369- MW112383 | MW112482- MW112496 | MW112595- MW112609 |
| 2017XZ006 | 10 | MW112271- MW112280 | MW112384- MW112393 | MW112497- MW112506 | MW112610- MW112619 |
| 2017XZ008 | 6 | MW112281- MW112286 | MW112394- MW112399 | MW112507- MW112512 | MW112620- MW112625 |
| GS201601 | 15 | MW112287- MW112301 | MW112400- MW112414 | MW112513- MW112527 | MW112626- MW112640 |
| QH201405 | 3 | MW112302- MW112304 | MW112415- MW112417 | MW112528- MW112530 | MW112641- MW112643 |
| SC201601 | 10 | MW112305- MW112314 | MW112418- MW112427 | MW112531- MW112540 | MW112644- MW112653 |
| SC201602 | 15 | MW112315- MW112329 | MW112428- MW112442 | MW112541- MW112555 | MW112654- MW112668 |
| YN201601 | 15 | MW112330- MW112344 | MW112443- MW112457 | MW112556- MW112570 | MW112669- MW112683 |
| GZ201601 | 15 | MW112345- MW112359 | MW112458- MW112472 | MW112571- MW112585 | MW112684- MW112698 |
